# Beyond chromosomes: exploring the diverse functions of extrachromosomal circular DNA

**DOI:** 10.1016/j.jare.2025.10.005

**Published:** 2025-10-10

**Authors:** Yaling Chen, Jian Wang, Xudong Liu, Xinxin Li, Zhuanjian Li, Hui Li

**Affiliations:** a Guangxi Key Laboratory of Animal Breeding, Disease Control and Prevention, College of Animal Science and Technology, Guangxi University, Nanning 530005, China; b College of Animal Science and Technology, Henan Agricultural University, Zhengzhou 450002, China; c Department of Liver Diseases, The Affiliated Ruikang Hospital of Guangxi Traditional Chinese Medicine University, Nanning 530011, China

**Keywords:** eccDNA, Enhancer, Transcription, Research methodology

## Abstract

Extrachromosomal circular DNA (eccDNA) is defined as a form of circular DNA that is widespread across various biological contexts and exists independently of chromosomal structures. The current distribution of total eccDNA identified in both healthy and tumor tissues shows genomic coverage ranging from 1.18% to 30.73%. EccDNAs are double-stranded, covalently closed, circular DNA molecules that originate from mechanisms such as somatic rearrangements, genomic instability, and hypertranscription. EccDNA possesses an open chromatin structure, which can participate in transcriptional regulation across the entire genome and mediate extensive interactions between eccDNAs and between eccDNA and chromosomes. EccDNAs vary from a few dozen base pairs to megabases (Mb) and may include anything from small non-coding sequences to entire genes and regulatory elements. Current research methods limit the ability to knock down or knock out eccDNA like genomic DNA. Overexpressing individual eccDNAs is also challenging, making it difficult to explore their molecular function. This review examines the underexplored potential functions of eccDNA, with particular emphasis on its role as an enhancer and its involvement in the production of transcribed mRNAs and non-coding RNAs (ncRNAs). In addition, we provided a method for in *vitro* synthesis of eccDNA and a method for generating eccDNA in cells based on CRISPR/Cas9 technology, which provides a research foundation for elucidating its biological functions and mechanisms of action. Finally, this paper presents innovative prospects from a forward-looking perspective regarding the functional mechanisms and potential applications of eccDNA in various biological scenarios.

## Highlights

• Circular topology of eccDNA underpins its unique regulatory functions.
• EccDNA exhibits an open, transcriptionally active chromatin state.
• Acts as a potent enhancer to directly drive gene expression.
• EccDNA transcription produces novel fusion non-coding RNAs.
• Effective tools play a key role in eccDNA mechanism research.

## Graphical abstract

**Figure:**
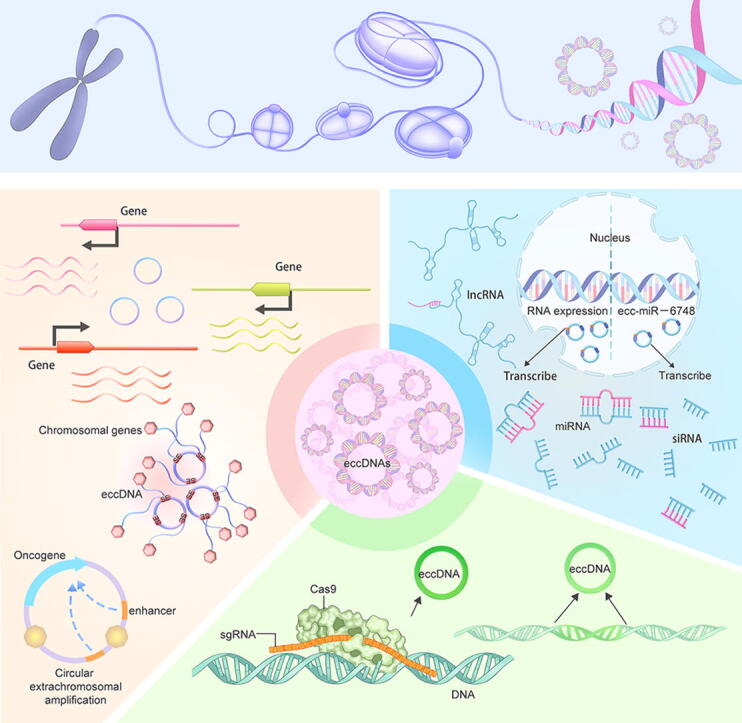

## Introduction

Extrachromosomal circular DNA (eccDNA) represents a distinct category of circular DNA molecules in eukaryotic organisms, capable of harboring supplementary genetic information that is distinct from the nuclear double-helix DNA [[1], [2], [3], [4]]. EccDNA predominantly resides within the nucleus; however, it is also present in the cytoplasm [[5], [6]]. Smaller fragments of eccDNA have been identified in extracellular locations, including exosomes [7,8], blood [[9], [10], [11], [12]], and urine [[13], [14], [15], [16]], initially identified in tumor cells [[17], [18], [19], [20], [21]]. EccDNA is now known to be common in various eukaryotes, such as yeast [[22], [23], [24], [25], [26]], nematodes [27,28], Drosophila melanogaster [29,30], mammals [[31], [32], [33], [34]], and plants [[35], [36], [37]]. EccDNAs possess several distinctive characteristics, including their composition as double-stranded circular structures, high amplification rates, the absence of centromeres, a loose chromatin configuration, and elevated levels of H3K27ac [[38], [39], [40], [41], [42], [43]]. The unique loop-like structure and relaxed chromatin configuration of eccDNAs enhance their capacity to regulate transcription throughout the genome [[44], [45], [46]].

EccDNA is characterized by a distinctive loop structure that imparts resistance to nucleases, thereby enhancing its stability and conferring unique biological properties in comparison to linear DNA [47,48]. These features facilitate the specific and stable inheritance of eccDNA, promoting adaptation to a wide range of physiological and pathological changes within organisms and contributing to the evolution of resistance and adaptation in both plant and animal species. In plants, eccDNA is integral to the amplification and transfer of herbicide resistance genes in crop weeds, thereby expediting the development of resistance and adaptation to glyphosate [49]. Under nitrogen-limiting conditions in the yeast, there is a significant increase in eccDNAs containing the ADP-ribosylation factor GTPase activating protein 1 (GAP1) gene, which facilitates environmental adaptation. In cancer cells, eccDNA frequently carries oncogenes and drug resistance genes, allowing for rapid replication and accumulation that confer a significant survival advantage to tumor cells [19,[50], [51], [52]]. In addition, eccDNA has been detected in the germline of Caenorhabditis elegans, suggesting its potential to be inherited as genetic material across generations [28]. It is noteworthy that eccDNA exhibits significant genomic variation potential and evolutionary significance in germline. It can be reintegrated into chromosomes, causing insertion mutations, structural rearrangements and other genetic variations, thus directly promoting genome diversity [53].

**Fig. 1:**
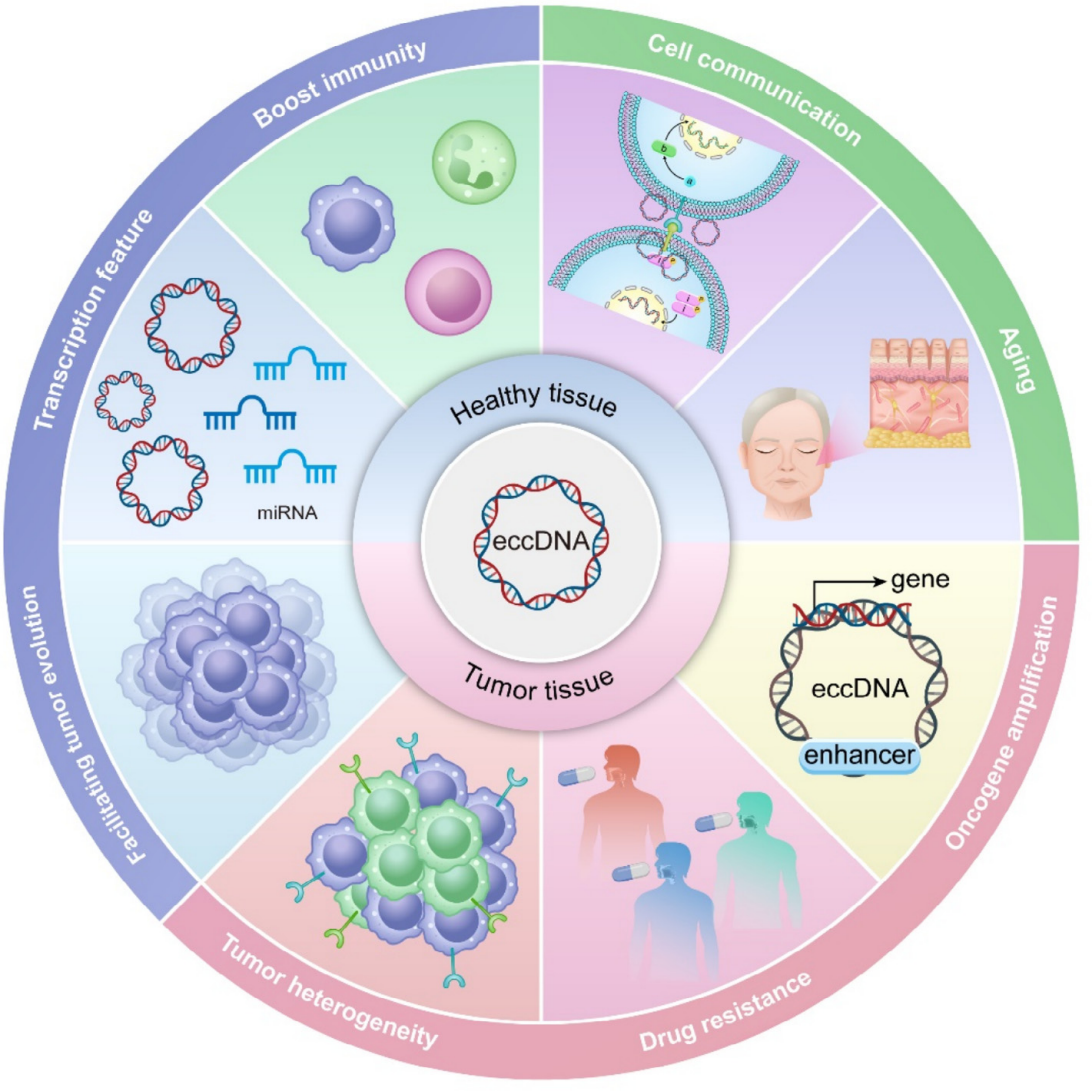
**Biological functions of EccDNA. Modulation of innate immunity:** EccDNA acts as a potent innate immune stimulator in a cyclic structure-dependent manner, primarily mediated via the STING pathway. It can also be released as a product of NETosis to activate inflammatory responses through pattern recognition receptors (e.g., TLR, *STING*, or *AIM2*). **Mediating intercellular communication:** Owing to its capacity to carry genetic information, high stability, and mobility, eccDNA serves as a potential messenger for intercellular communication and signal amplification. **Senescence:** In yeast, eccDNAs derived from ribosomal DNA or the *CUP1* gene accumulate with age and promote cellular senescence. (Note: The role of eccDNA in mammalian cellular senescence remains hypothetical, and direct evidence is still needed.) **Oncogene amplification and high expression:** Their circular architecture and open chromatin state enable high transcriptional activity, facilitating massive oncogene expression that promotes tumor progression, metastasis, and poor prognosis. **Tumor heterogeneity and drug resistance:** EccDNA is unevenly distributed to daughter cells during cell division, leading to intratumoral genetic heterogeneity and drug resistance. **Transcription of non-coding RNAs:** Certain small eccDNAs (e.g., microDNAs) can be transcribed into functional miRNAs or siRNA-like molecules, which silence their cognate gene targets.

The role of eccDNA is increasingly investigated due to technological advancements, highlighting its significance across various biological domains **(**Fig. 1**)**. EccDNA is prevalent in numerous malignancies including glioblastoma [54], neuroblastoma [55], medulloblastoma [56], lung [57], liver [58], gastric [59], breast [60], ovarian [61], and prostate [62] cancers, playing a pivotal role in cancer development. Moreover, eccDNA is linked to age-related ailments like Werner syndrome, amyotrophic lateral sclerosis (ALS), and type 2 diabetes mellitus (T2DM), potentially exacerbating disease progression through genome stability alterations [63]. This positions eccDNA as a promising prognostic biomarker and therapeutic target for diseases. This study delves into the multifaceted biological roles of eccDNA. Firstly, eccDNA housing enhancer elements can function as “mobile enhancers” [46,64,65], dynamically modulating gene expression throughout the genome, or intensifying regulatory impacts via “eccDNA hubs” [43,66,67]. Particularly, the co-existence of oncogenes and enhancers within the same loop molecule can profoundly reshape gene regulatory networks through amplification effects [20,[67], [68], [69]]. Secondly, the plausible transcriptional functions and mechanisms of eccDNA containing multiple RNA coding sequences (e.g., mRNA, circRNA, miRNA, and lncRNA) are examined, alongside the proposition of various hypotheses. Lastly, potential novel avenues for further exploration in eccDNA research are highlighted.

### Classification of eccDNA

A consensus has yet to be reached in the scientific community regarding a uniform definition and categorization of circular DNA **(**Table.1**)**. These molecules exhibit a wide range of sizes, spanning from tens of base pairs to multiple megabases [6,70]. Circular DNA is commonly classified using two primary methods: one based on molecular size, which distinguishes between extrachromosomal DNA (ecDNA) and eccDNA, and another method that classifies them according to the genetic components they contain. The classifications are as follows:

1) EcDNA

**Table 1:** The different types of eccDNA.

Classification		Description		Ref.
By length	EccDNA		Mostly < 100 kb; Aging; Enhancer; Cell communication; Stimulation of innate immune pathways; Expression of regulatory RNAs.	[9,139]
	EcDNA		1 Mb–3 Mb; Oncogene amplification and genetic heterogeneity.	[41,104]

By function	Telomeric circle		Integral multiples of 738 bp; Maintaining telomeres and proliferating.	[71,79,80]
	MicroDNA		Mostly between 200–400 bp; Producing miRNAs.	[2,82,88,140]
	SpcDNA (small polydispersed circular DNA)		100 bp-10 kb (occasionally > 10 kb); Causes genomic instability.	[78]
	Episome		Approximately 250 kb; Precursors for DM.	[73]
	Double minutes (DMs)		100 kb-3 Mb; Amplifying extrachromosomal gene.	[141]

By origin	Exon eccDNA		Produced in exonic regions.	[42]
	Intron eccDNA		Arises in the intronic region.	
	Repeat eccDNA		Derived from repeated sequences.	
	Repeat-intergenic eccDNA		Lies in non-coding regions and repeats.	
	Intergenic eccDNA		Arising from the intergenic region.	
	Transposable element (TE) / Promoter / Enhancer eccDNA		Carrying regulatory elements.	
	Whole-gene eccDNA		Carrying the full gene.	[43]

In oncology, ecDNA is a large circular DNA molecule in cancer cells that exists independently of chromosomes [18]. Because of its large size, ecDNA can carry multiple genes, even complete proto-oncogenes [20]. This DNA is widely found in a variety of human cancers [10,71]. As cells divide, ecDNA is not passed on according to Mendelian inheritance, but is randomly distributed among daughter cells, rapidly increasing the number of copies of oncogenes [41]. This mechanism accelerates the formation of tumor heterogeneity and malignant evolution [39], and is closely related to increased invasiveness [17], treatment resistance and poor prognosis of patients [19,72]. Morphologically, ecDNA can be observed by light microscopy and can be divided into two main categories: episomes [73] and double minutes (DMs) [74,75]. Additions are located in the cytoplasm and may be integrated into chromosomes, while double microsomes generally occur in pairs and belong to circular DNA structures lacking centromere and telomere. About a third of ecDNA exists as double microsomes, the study showed.

2) EccDNA

EccDNA is typically smaller than ecDNA and is prevalent across various eukaryotic cells and tissues, including both normal and tumor environments [9,76]. Current research categorizes eccDNA into small polydisperse circular DNA (spcDNA) [77,78], t-circle [79,80], extra-chromosomal rDNA loop (ERC) [38], and microDNA [81,82], each with distinct structural and functional characteristics. SpcDNA, or small polydisperse circular DNA, ranges from hundreds to thousands of base pairs and often originates from genomic repeat sequences, though its formation mechanism remains unknown. It is more frequent in genetically unstable cells or tissues, indicating a strong link to genomic instability [83]. T-circles, composed entirely of telomere repeats, are extrachromosomal telomere structures. While they can mimic telomerase by extending telomere repeats to delay replicative shortening, their formation mechanism is unclear and is generally associated with cellular aging [84]. ERCs are circular DNA molecules that harbor rDNA sequences and are considered a form of spcDNA [85]. MicroDNA, on the other hand, refers to small circular DNA fragments of approximately 200–400 bp in length, originating from non-repetitive genomic regions such as 5′ untranslated regions (UTRs), exons, or CpG islands. Due to their diminutive size, microDNA typically do not encompass complete genes and lack the capacity to encode proteins [82]. However, they can contribute to the regulation of gene expression by generating regulatory small RNAs, including microRNAs or novel siRNA-like transcripts.

3) Additional Types

The diversity of genetic elements carried by eccDNA is extensive. The specific structure of eccDNA is determined by the genomic breakpoints and can encompass a wide range of forms, from short non-coding sequences to entire genes. Depending on their origin and composition, eccDNA can be categorized into various types, such as whole-genome, exon-specific, intron-specific, repetitive sequence-specific, repetitive intergenic, intergenic, transposable element (TE), and promoter/enhancer types [42]. TE and promoter/enhancer types of eccDNA are particularly noteworthy for their ability to undergo self-amplification through circularization units and integrate into other eccDNAs to form larger circular structures, thus termed as “function-enhanced eccDNA” [42,86]. Naming eccDNA based on the specific sequences it carries is more appropriate as its functionality is largely dictated by these elements. Hence, this naming convention is preferred. Furthermore, extensive research has revealed the ubiquitous presence of eccDNA in nearly all eukaryotic organisms, not restricted to tumor tissues but also found in normal tissues. It is important to note that while large-fragment eccDNA can be identified in healthy tissues, small-fragment types are also present in tumor tissues. Therefore, defining eccDNA solely based on tissue origin or fragment size is not entirely accurate.

In this study, we refer to these as eccDNA and further explore their functional characteristics based on the elements they contain.

### Factors associated with eccDNA biogenesis

**Fig. 2:**
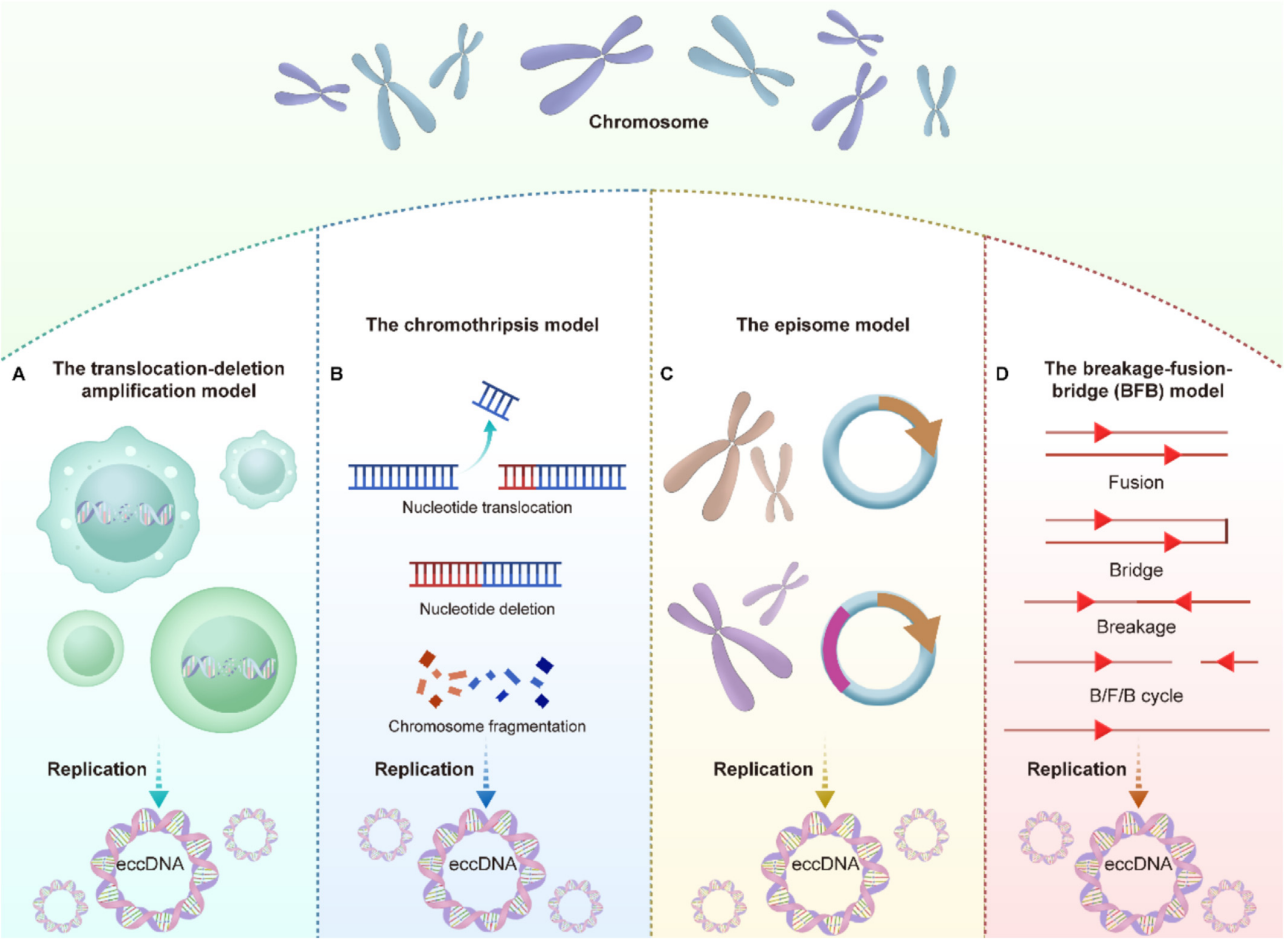
**Models of eccDNA biogenesis. (A) The translocation-deletion-amplification model.** Chromosomal translocation initiates gene rearrangements. The DNA fragments near the breakpoints can then be deleted, amplified, and circularized to form eccDNAs. **(B) The chromothripsis model.** Catastrophic chromosomal shattering generates numerous acentric DNA fragments, a subset of which may be ligated into circular eccDNAs. **(C) The episome model.** EccDNAs can be generated directly through the excision and circularization of genomic segments, often via over-replication or recombination. **(D) The breakage-fusion-bridge (BFB) cycle model.** The cycle begins with telomere loss, leading to the fusion of sister chromatids and the formation of a dicentric chromosome. During anaphase, the dicentric chromosome forms a bridge that breaks upon cell division, potentially releasing linear and circular DNA fragments.

The exact mechanism behind eccDNA formation remains elusive, with several hypotheses proposed **(**Fig. 2**)**. Chromosomal instability and replication stress are thought to be crucial in eccDNA production, particularly in cancer cells [6]. EccDNA may arise from DNA replication processes, such as the excision of chromosomal loops or mis-ligation of DNA fragments during replication pauses [87]. Additionally, the nonhomologous end joining (NHEJ) pathway, active during DNA replication and double-strand break repair, is considered significant for eccDNA formation [88,89]. Replication origin deletion and chromosomal rearrangements may lead to chromosome breakage, indirectly facilitating eccDNA production [89]. External factors like radiation and drug treatments can induce DNA fragmentation, further promoting eccDNA formation [90,91]. Short repeat sequences might mediate genetic recombination in this process [56]. Findings suggest that eccDNA production relies on DNase γ-catalyzed apoptotic DNA fragments and DNA ligase 3 (Lig3)—catalyzed cyclization. Recently, a new model suggests that the YY1 protein mediates DNA circularization and ligation by Lig3 in an acidic microenvironment created by PARylation modification, potentially independent of classical DNA double-strand breaks [92]. In summary, the eccDNA formation mechanism is diverse and complex, with ongoing research uncovering new formation pathways.

1) High Transcriptional Activity

EccDNA is widely present in the somatic cell genome, and its distribution is non — random, showing obvious genomic location preferences. More than 50 % of eccDNA is derived from gene or pseudogene regions, and is significantly enriched in coding regions in particular, suggesting that its formation may be closely related to key cellular processes such as gene transcription and DNA repair. Studies have shown that the formation of eccDNA is significantly correlated with high transcriptional activity. For example, in the human titin gene (*TTN*), a narrow region exhibits an abnormally high density of eccDNA formation [9]. Since *TTN* is highly expressed in muscle tissues and participates in the regulation of resting muscle plasticity, the enrichment of eccDNA in this region provides strong evidence that high transcription promotes eccDNA generation. In addition, broken transcripts and transcripts of the Huntington's disease — related gene *HIP1* were also detected in the *TTN* region, indicating that eccDNA can not only be derived from non — transcribed regions, but some types can also remain in the nucleus and have transcriptional activity. This type of eccDNA with transcriptional potential may affect cell phenotypes by producing truncated or full — length transcripts and even participate in disease development. Mechanistically, eccDNA tends to form in highly transcribed regions and may play a role of “signal hijacking” or “amplification” in gene expression regulation and disease progression through its transcriptional ability. Multiple lines of evidence consistently indicate that eccDNA — positive regions are positively correlated with open chromatin (such as compartment A), compartment boundaries, expressed genes, exons, introns, and histone modifications (such as H3K27ac, H3K4me1), further supporting that its formation is regulated by transcriptional levels and is tissue — specific [93]. Especially under high — transcription conditions, the number of eccDNA shows a logarithmic increase, indicating that highly expressed genes are more likely to be its sources [17].

2) Gene Length and Chromosome Size

Gene length significantly influences eccDNA formation, particularly in longer genes. Mechanistically, genes with greater physical spans are more prone to DNA breaks and abnormal recombination during replication or transcription, facilitating eccDNA formation. Furthermore, the extended transcription time of long genes exposes DNA templates to endogenous damage for longer periods, increasing eccDNA production likelihood. For instance, *TTN* genes and regions exceeding 300 kb generate more eccDNA than shorter genes [9]. EccDNA formation often coincides with large chromosomal segment deletions. In the *DAZ4* gene [94], a deletion matching the eccDNA break site was observed, and eccDNA was frequently produced in high—frequency regions like *HLA*, *KIR*, and *SERF1A*_*SMN2* [95], suggesting shared molecular mechanisms. The study also demonstrated a positive correlation between eccDNA quantity and chromosome length in mouse models. At the gene level, eccDNA production rose with gene length, underscoring the pivotal role of gene length in its formation. Additionally, these genes are significantly enriched in biological processes such as synaptic function, cell connectivity, adhesion, and neural development. These findings confirm the broad impact of gene length and suggest that eccDNA formation may participate in specific physiological functions.

3) Chromosome gene density

In gene-rich chromosomal regions, eccDNA is more prone to formation, particularly in chromosomes with high gene density [9]. The rate of eccDNA generation per unit length in these regions surpasses that in gene—sparse chromosomes and the overall average [14,96]. Studies have shown a positive correlation between the frequency of eccDNA formation and coding gene density, suggesting that these regions create a conducive environment for eccDNA generation [9,20]. Notably, no significant association was found between eccDNA formation and pseudogenes, short variant genes, or other non-coding genes. This selective correlation implies that coding genes possess unique biological characteristics, such as high transcriptional activity or specific chromatin structures, which likely play a pivotal role in eccDNA formation [78]. Thus, while coding gene density is linked to eccDNA production, the primary driver may not be transcriptional activity per se but rather other underlying biological attributes of the coding regions that are yet to be fully elucidated.

4) Elevated GC content

In terms of the origin mechanism, eccDNA shows an obvious tendency to be generated from genomic regions with high GC content, and its sources are widely distributed across multiple segments of the human genome [59,97]. At the structural level, GC base pairs in high—GC regions are connected by three hydrogen bonds. Compared with AT pairs maintained by only two hydrogen bonds, they possess higher thermal stability and chemical resistance, which contributes to the formation of more stable DNA segments. This stability promotes the generation of eccDNA in two ways: on one hand, it enhances the ability of linear DNA fragments to resist nuclease degradation, prolonging their retention time in the nucleus and providing the possibility for circularization; on the other hand, it helps maintain the spatial conformation conducive to the intramolecular ligation reaction, thereby improving the circularization efficiency [6]. Notably, high-GC regions often coexist with functionally active chromatin structures such as gene-dense regions, active promoters, and CpG islands. These regions typically exhibit a highly open chromatin state, with DNase I hypersensitive sites and specific histone modifications (e.g., H3K4me3 and H3K27ac), and are more susceptible to mechanical stress and metabolic disturbances during transcription and replication. The accumulation of R-loops and frequent topoisomerase-mediated breakage events in these regions significantly increase the risk of double-strand breaks, which can then promote the formation of eccDNA through mechanisms such as microhomology-mediated end joining (MMEJ) or break-induced replication [88]. Therefore, high GC content not only enhances the physical stability of DNA but also indicates the functional activity and instability of the region in the genome. It also reflects its central role in gene regulation and implies that it is more prone to recombination under stress conditions. Overall, there is a multi-level close connection between the formation of eccDNA and high-GC regions at the molecular level. This not only reveals the structural basis for its occurrence but also suggests that eccDNA may serve as a dynamic element in the gene regulatory system and play a crucial role in the occurrence and development of diseases such as cancer.

### EccDNA mediates enhancer-driven gene transcription

1) As Mobile Enhancer

**Fig. 3:**
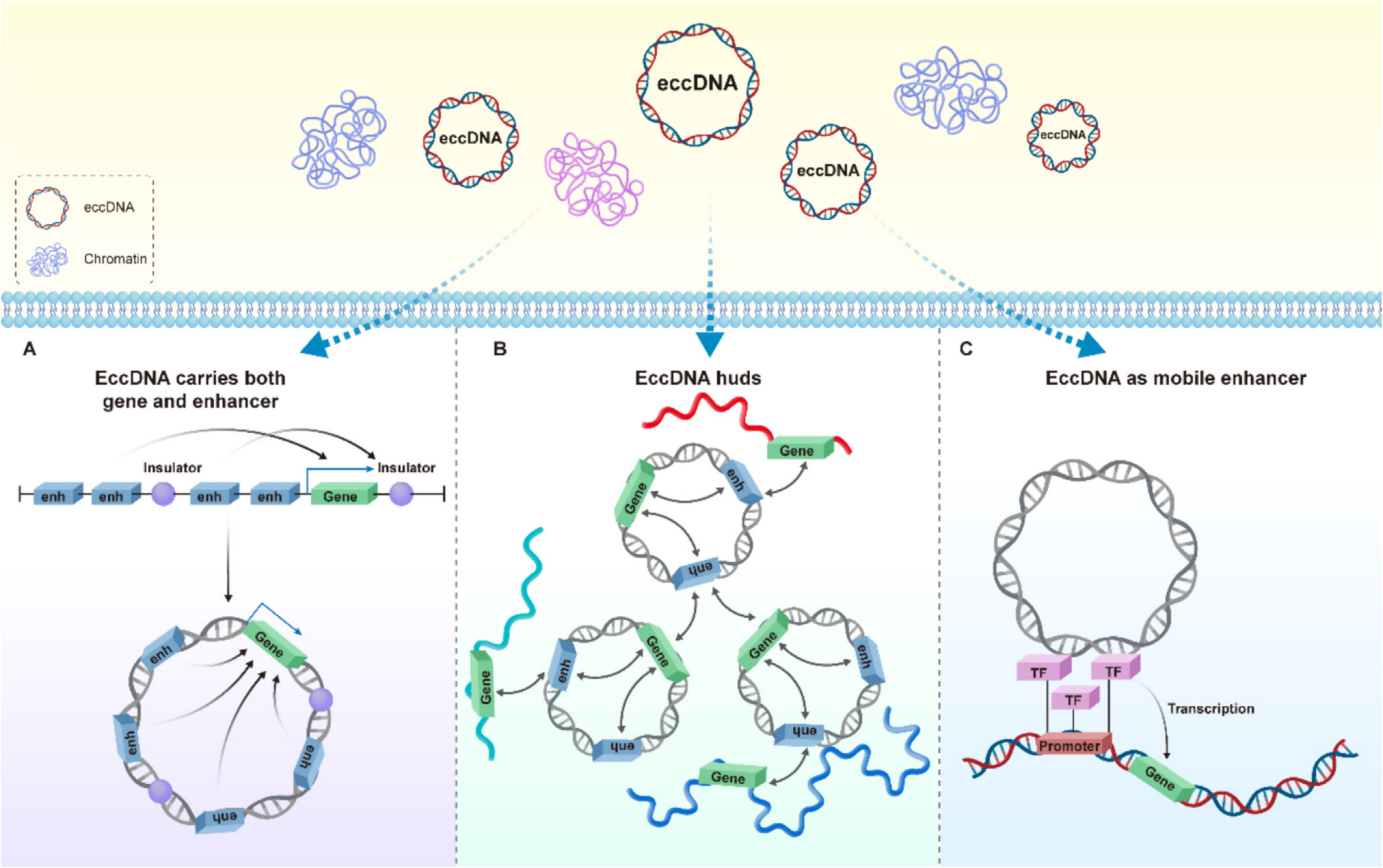
**Enhancer Function of EccDNA. (A)** The co-amplification of oncogenes alongside neighboring enhancers on eccDNA serves to strengthen existing regulatory associations and introduce new ones. These enhancers, which are primarily super-enhancers or classical enhancers, are characterized by a high density of H3K27ac and are targeted by tissue-specific transcription factors. This results in the activation of both nearby and distant oncogenes through *trans*-regulatory mechanisms. **(B)** EccDNA hubs support efficient transcription through spatial aggregation, dependence on BET proteins to maintain structural stability, and promotion of intermolecular interactions between enhancers and genes. Multiple active enhancer elements are clustered within hubs to form a structure known as a “combinatorial enhancer platform”. This platform can be regarded as a highly functional enhancer hub that efficiently recruits transcriptional machinery (e.g., RNA polymerase II) and transcription factors to drive abnormally high levels of transcription of the oncogenes it carries. **(C)** EccDNA exhibits enhanced mobility within the nucleus. eccDNA molecules carrying potent enhancers can relocate to chromosomal regions and transactivate oncogene transcription ectopically via enhancer-promoter interactions.

EccDNA is a special type of “mobile enhancer” [65,98]. Unlike ordinary enhancers that are confined within chromosomes, it can move freely in the nucleus **(**Fig. 3**C).** This is because eccDNA has a circular structure without a centromere, thus being unrestricted by chromosomal spatial constraints. This ability to move freely enables it to influence other genes at a distance. Research has shown that eccDNA frequently interacts with genes on multiple chromosomes, and the frequency of such cross — chromosomal interactions is much higher than that of conventional chromosomal DNA. Even after excluding the influence of its high copy number, eccDNA still exhibits a stronger tendency for cross — chromosomal interactions, indicating that this ability stems from its circular structure itself, rather than simply being due to a larger quantity. There are a large number of active regulatory elements on eccDNA, and their interactions with chromosomes are concentrated in non — coding regions with the characteristics of super — enhancers [99]. EccDNA shows high affinity for the promoter regions of chromosomal genes and, by virtue of its mobility, “delivers” the potent enhancers/super — enhancers it carries to different positions on different chromosomes, interacting with the promoters of distal target genes in a trans — acting manner. This mechanism can lead to the high — frequency transcriptional activation of oncogenes on chromosomes and cause a significant enrichment of H3K27ac in the target regions. Notably, a single eccDNA molecule can carry multiple regulatory elements and genes simultaneously, enabling the co — aggregation of multiple oncogenes through super — enhancers to form an efficient transcriptional “factory”, thereby coordinating their co — activation [43]. Although eccDNA has only a few contact points, it dominates a large proportion of chromosomal interactions, showing high specificity. This indicates that eccDNA does not interact randomly but is a regulatory element with precise functions that can actively influence genomic interactions [46,100]. The existence of eccDNA challenges the traditional view of the static nature of chromatin territories, suggesting that the genomic spatial structure is dynamic [65]. Mobile genetic elements can actively and extensively reconstruct the gene regulatory network, which becomes a key mechanism for cancer cells to acquire plasticity. From a therapeutic perspective, targeting eccDNA itself or the specific chromatin interactions it mediates (e.g., targeting the SEs on it) holds promise for the development of novel tumor treatment approaches that are more specific and promising than traditional strategies.

2) Gene- and Enhancer-Bearing eccDNA

During tumorigenesis, gene amplification typically manifests as chromosome duplication or eccDNA formation **(**Fig. 3**A)**. EccDNA can harbor complete oncogenes, with its chromatin in a highly accessible state, often accompanied by various enhancers, thereby significantly boosting transcriptional activity [20]. This suggests that eccDNA-mediated oncogene overexpression is not merely due to increased copy number but is intricately linked to its elevated transcriptional potential [45]. Notably, noncoding elements such as enhancers on eccDNA play a pivotal role in oncogene regulation. When oncogenes and adjacent enhancers are co-amplified on the same eccDNA, they can reinforce existing regulatory interactions or establish new networks, accelerating tumor progression [19]. Evidence suggests that the co-amplification of enhancers and oncogenes in multiple tumor types may not be random but reflects significant tissue specificity. For instance, *EGFR* is co-amplified with a specific enhancer in glioblastoma, and a similar mechanism is observed for *ERBB2* in breast cancer, illustrating this biological phenomenon [68]. In neuroblastoma, *MYCN* is co-amplified with a 3′ superenhancer active in fetal adrenal tissue, which *MYCN*, *TWIST1*, and *GATA3* can bind [101]. Conversely, in Wilms tumor, the MYCN oncogene is co-amplified with a 5′ superenhancer specific to embryonic kidney tissue, reliant on *SIX1* and *SIX2* for recognition [103]. This indicates that enhancer selection on ecDNA is non-random, intricately linked to the tumor's tissue origin, and may represent an aberrant mimicry of developmental programs within the tumor microenvironment.

From a mechanistic perspective, the co-amplified enhancers are predominantly super-enhancers or classical enhancers, characterized by a high density of H3K27ac modifications. These enhancers can be recognized by tissue-specific transcription factors, which can then activate distant or adjacent oncogenes in a *trans*-acting manner. The physical proximity between the enhancer and oncogene further establishes a highly coordinated regulatory unit, significantly enhancing the transcriptional efficiency of the oncogene [65]. This intricate regulatory architecture provides valuable insights into the mechanisms underlying tumorigenesis. Additionally, in multiple myeloma, the transcription factor *POU2F2* can bind to the enhancer region of eccANKRD28 and interact with *RUNX1/RUNX2* motifs, forming protein complexes that remotely activate the transcription of *IRF4*, *JUNB*, *IKZF3*, and other oncogenes, thereby promoting the emergence of drug resistance phenotypes [102]. This discovery expands our understanding of the complexity inherent in the eccDNA regulatory network.

3) Formation of eccDNA hubs

In cancer cells, eccDNAs containing oncogenes tend to aggregate, forming structures termed eccDNA hubs [43] **(**Fig. 3**B)**. These hubs arise from the dense clustering of dozens to hundreds of eccDNA molecules within specific nuclear regions post-cell division or DNA damage, displaying a pronounced propensity for coalescence. Oncogenes located on eccDNA hubs demonstrate heightened transcriptional activity and likelihood compared to those on chromosomal DNA [67,20]. The collective arrangement and increased abundance of eccDNA amplify the chances of a single eccDNA molecule initiating oncogene transcription. Consequently, the level of eccDNA aggregation closely correlates with oncogene transcriptional activity. Morphologically, these hubs surpass chromosomal segments of equivalent size and can function as composite enhancer platforms. By orchestrating the reorganization of intermolecular enhancers, they propel the robust expression of oncogenes, fostering novel enhancer-promoter interactions that bolster oncogene transcription [104]. The uneven distribution of enhancers across oncogenes further exacerbates cellular diversity [105]. Moreover, eccDNA hubs may encompass long-range transcriptional regulatory elements situated on distinct eccDNA molecules, thereby influencing the expression of distant genes [106]. This revelation not only elucidates the mechanism through which the selective aggregation and regulatory reconfiguration of eccDNA stimulate transcription but also unveils potential targets for advancing cancer therapeutic strategies.

### EccDNA-Mediated transcription of coding and non-coding RNAs

1) EccDNA-Mediated Transcription of mRNA

In cancer research, eccDNA has been confirmed to serve as a transcriptional template. These circular molecules often carry multiple oncogenes and are unevenly distributed to daughter cells during cell division, resulting in significant cell-to-cell heterogeneity in oncogene copy number [17]. Due to their circular structure and more open chromatin state, eccDNA molecules confer a transcriptional advantage and can drive tumor proliferation more efficiently than their linear counterparts [107]. The oncogenes they carry frequently exhibit exceptionally high transcriptional activity, with some of them ranking among the top 1 % of the most highly expressed genes in the tumor transcriptome. For example, in pancreatic ductal adenocarcinoma (PDAC), eccDNA significantly amplifies the oncogenic potential of *MYC* by acting as both a carrier and an amplifier [108]. Key mechanisms include substantially increasing gene copy number, forming specialized nuclear transcription hubs, and enabling dynamic responses to environmental stress. Similarly, in neuroblastoma, eccDNA promotes tumor progression and induces drug resistance through amplification of the MYCN oncogene [96]. Beyond oncogenes, eccDNA can also carry various immune regulatory genes. Thus, it not only accelerates tumor progression via oncogene amplification but may also help tumor cells evade immune surveillance through immune-related genes, thereby playing multifaceted roles in cancer progression and the remodeling of the tumor immune microenvironment [97,109]. Although other pathways can also upregulate oncogenes in cancer, gene amplification remains the core mechanism through which eccDNA exerts its oncogenic functions.

2) Transcription generates circRNA

Some viewpoints suggest that eccDNA may be involved in the generation of circRNA [110,16]. Although this hypothesis has not been fully confirmed by experiments, it is reasonable from the perspective of molecular mechanisms. Both eccDNA and circRNA are circular − structured molecules, both have high transcriptional activity, and despite different generation mechanisms, their circular conformations are similar [42]. It is speculated that eccDNA may affect the transcription process of Pol II (such as causing template slippage or continuous transcription), making it easier to produce pre − circularized transcripts, which can form circRNA through backsplicing [111,112]. In addition, eccDNA is derived from chromosomal DNA and is widely distributed in the genome, capable of carrying complete genes and their regulatory elements. They aggregate in certain regions to form “eccDNA hubs”, which may significantly increase the transcription frequency and intensity in these regions [66]. Meanwhile, eccDNA is usually in a highly open chromatin state, which is closely related to active transcription and also creates favorable conditions for circRNA generation. eccDNA also has the ability to integrate sequences from different chromatin regions, forming new combinations that do not originally exist in chromosomes, thereby increasing the chances of potential circRNA generation. In terms of the formation mechanism, circRNA relies on backsplicing, which usually requires complementary pairing of flanking introns or the assistance of RNA — binding proteins (RBPs) [[113], [114]]. If the gene sequences carried by eccDNA contain introns, they may provide the key flanking structures required for the backsplicing of their transcripts. Some studies in Arabidopsis suggest that some circRNAs may be derived from the transcription of eccDNA, and their boundaries often carry 4–––12 bp inverted repeat sequences [110]. This sequence feature is conceptually similar to the reverse − complementary mechanism required for circRNA formation, providing preliminary sequence − based evidence for the above hypothesis. However, at present, these understandings are still reasonable speculations and require more experimental verification.

3) Transcription generates miRNA and siRNA

**Fig. 4:**
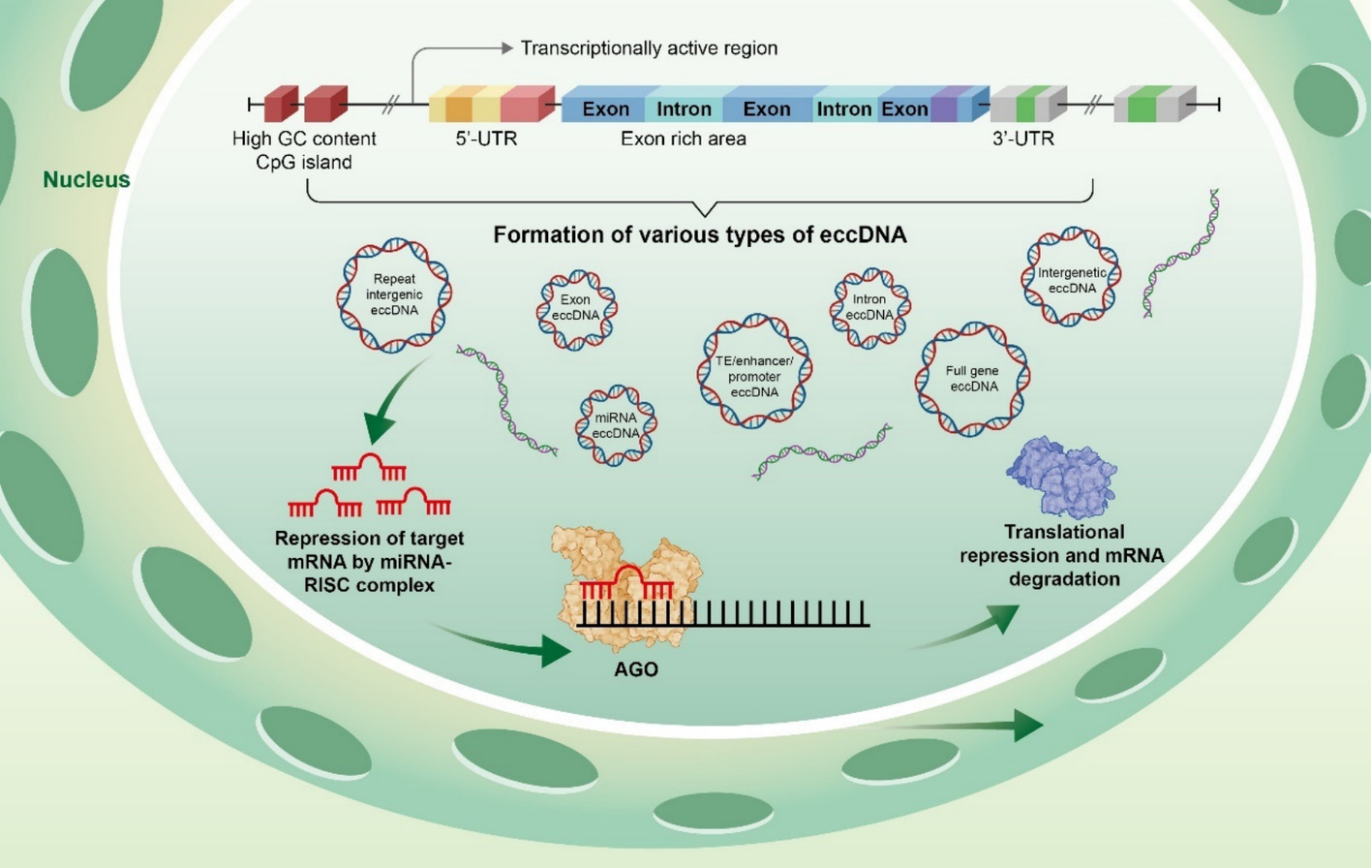
**Model of miRNA-mediated gene silencing via eccDNAs.** MicroDNA and other eccDNAs harboring miRNA precursor sequences can be transcribed into primary miRNAs (pri-miRNAs), which are subsequently processed into mature miRNAs. These miRNAs are incorporated into the RNA-induced silencing complex (RISC) to mediate post-transcriptional silencing of complementary endogenous mRNA targets.

As a type of eccDNA, microDNA exhibits significant transcriptional activity **(**Fig. 4**)**. Even in the absence of typical promoters, it can efficiently transcribe to generate microRNAs (miRNAs) and siRNA-like molecules with regulatory functions [58,82]. These RNA molecules can be equally transcribed from double-stranded DNA in a non-strand-specific manner, with a maximum gene expression inhibition efficiency of up to 80 %, and can exist in single-stranded or double-stranded forms. Some microDNAs themselves carry miRNA coding regions and can directly transcribe to generate mature miRNAs, participating in the gene regulation process. These microDNA molecules are often enriched in gene regions, and some even originate from exon sequences. Studies have shown that circular DNA derived from exons can transcribe to generate novel siRNAs, which target and inhibit the expression of their parental genes. The discovery of the binding of RNA polymerase subunits to microDNA provides direct evidence for its transcriptional activity. Additionally, a type of single-stranded circular DNA with a length of 34–89 bp can be transcribed through the rolling circle mechanism in vivo and in *vitro*. RNA polymerase synthesizes repetitive RNA units along the circular template, and subsequent processing can generate small RNAs with regulatory functions. Similar phenomena also exist in other organisms. For example, double-stranded circular DNA derived from transposons in Paramecium tetraurelia can also transcribe regulatory small RNAs [82]. Other studies have shown that larger fragments of eccDNA can even encode oncogenic miRNAs. These findings collectively indicate that eccDNA is an important class of non-classical genetic elements that can widely participate in the fine regulation of gene expression by producing various regulatory RNAs [[115], [116]]. This not only expands our understanding of the gene expression mechanism but also provides a new perspective for understanding genomic plasticity and transcriptional instability. Some gene regulatory pathways may be more flexible and complex than previously thought, making them more adaptable to the regulatory needs of cells in changing environments.

4) Transcription generates lncRNA

EccDNA can be derived from multiple genomic regions, including gene coding regions, enhancers, repetitive sequences, intergenic regions, or introns. Of particular interest are large eccDNAs derived from intergenic or intronic regions (which are common in cancer), as these genomic regions are also the primary sources of lncRNAs [117. Consequently, such eccDNAs frequently carry sequences capable of encoding lncRNAs. The circular structure of eccDNA, often coupled with active histone modifications (e.g., H3K27ac), helps maintain an open chromatin state, thereby enhancing its transcriptional activity [65]. Circularization may also create atypical transcription start sites near junction sites, potentially because it alters the local chromatin environment or disrupts topologically associating domains (TADs). This can expose previously silent sequences and initiate atypical transcription, which may further lead to the production of chimeric transcripts or novel regulatory elements with potential carcinogenic effects. In cancer, eccDNA is frequently associated with the amplification and overexpression of oncogenes. The lncRNAs transcribed from eccDNA may function as competing endogenous RNAs (ceRNAs, or molecular sponges), which could interfere with normal RNA regulatory networks and indirectly promote tumor progression, although direct experimental evidence for this mechanism remains limited [118].

### Effective tool for studying eccDNA roles

To date, numerous advanced studies have been conducted focusing on the expression profiles of eccDNA [14,15,119,120]. Notably, multi-omics joint analyses have emerged as a prominent approach, with common methodologies including eccDNA-mRNA, eccDNA-whole genome sequencing (WGS) [121], eccDNA-miRNA, eccDNA-RNA N6-methyladenosine (m6A) modification, and eccDNA-assay for transposase-accessible chromatin using sequencing (ATAC-seq) analyses [120,122]. The availability of effective research tools significantly enhances the investigation of molecular mechanisms associated with eccDNA. This paper outlines 4 reliable methodologies for exploring the molecular mechanisms underlying eccDNA:

1) LAMA

**Fig. 5:**
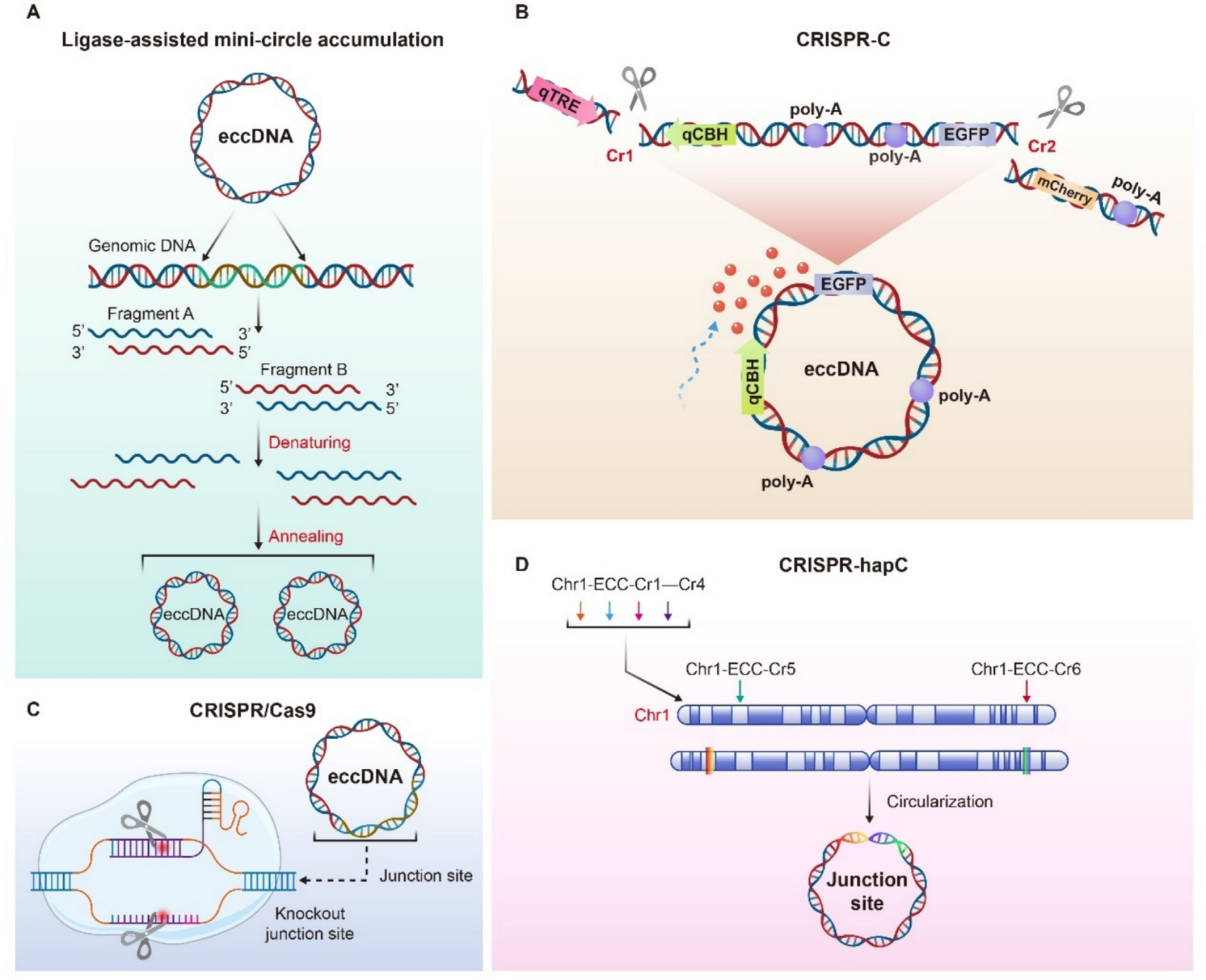
**Schematic illustration of strategies for eccDNA synthesis: in vitro versus intracellular approaches. (A) LAMA:** Flowchart of the LAMA method for in vitro assembly of artificial eccDNA. Two complementary oligonucleotides (Fragment A and Fragment B, the latter being the reverse complement of Fragment A) are hybridized through annealing to form a nicked open-circle structure. The nick is subsequently ligated by DNA ligase to yield intact double-stranded eccDNA. **(B) CRISPR-C:** CRISPR-mediated eccDNA generation via dual CRISPR/Cas9 cleavage at sites flanking a chromosomal locus. Circularization is mediated by non-homologous end joining (NHEJ), reorienting an inverted promoter into its functional configuration to activate a fluorescent reporter gene. Successful eccDNA formation is detectable via fluorescence. **(C) CRISPR/Cas9:** The CRISPR/Cas9 system can be designed with sgRNAs targeting the eccDNA junction sequence, enabling modulation of eccDNA copy number through targeted cleavage and degradation. **(D) CRISPR-hapC:** CRISPR-hapC enables haplotype analysis by exploiting CRISPR-induced eccDNA formation. It directs double-strand breaks at specific genomic sites, promoting the linkage of two distal regions into a circular DNA molecule via cellular repair mechanisms, thereby capturing haplotype-specific information.

Ligase Assisted Mini-circle Accumulation (LAMA) is one of the commonly used methods for constructing eccDNA at present **(**Fig. 5**A)**. This method first designs and chemically synthesizes four oligonucleotides as starting materials based on the sequence of the target eccDNA. The basic principle is that after denaturation and annealing, these four oligonucleotides will self − assemble into two linear double — stranded DNAs with reverse complementary sequences, which will further cyclize to form a double − stranded circular intermediate with two nicks. Subsequently, the nicks are sealed by DNA ligase, ultimately forming a complete double − stranded circular DNA (i.e., the LAMA product). However, some incomplete products such as single — nicked circular DNA and linear DNA are often produced during the cyclization process [123]. Therefore, the final reaction product is a mixture containing various forms such as complete circular DNA and linear DNA, and usually needs to be separated and purified by gel electrophoresis to obtain the target eccDNA [58,124]. The main advantages of the LAMA method are high flexibility, relatively simple operation, and independence from cells. It can synthesize eccDNAs of different lengths in vitro and can be transfected into cells as efficiently as plasmids, with wide applications. However, its disadvantages are also obvious: low synthesis efficiency, limited yield, cumbersome experimental steps, and high reagent costs.

2) CRISPR-C

The technique for generating eccDNA utilizing CRISPR technology has been subsequently termed CRISPR-C. This designation pertains to the circularization of genes and chromosomes mediated by CRISPR within cellular environments, thereby enabling the production of endogenous eccDNAs of diverse lengths **(**Fig. 5**B)**. This is achieved through dual CRISPR cleavage at both the 5′ and 3′ termini of the eccDNA situated on a linear chromosome. This method is based on the principle of employing CRISPR technology to induce cuts at two separate loci on the same chromosome, facilitating the cyclisation of the intervening sequence through blunt-end ligation. By positioning an inverted promoter and a fluorescent protein gene at each terminus, cyclisation of the DNA molecule results in the spatial alignment of the promoter and the fluorescent protein gene, thereby initiating the expression of the fluorescent protein gene. Consequently, the occurrence of DNA cyclisation can be detected through the expression of the fluorescent protein. CRISPR-C's creation impacts the investigation of eccDNA mechanisms [107,125].

CRISPR-C is an efficient experimental technique that cuts specific genomic loci based on the CRISPR/Cas9 system and specifically induces the generation of specific eccDNAs through the cell's own repair mechanism. The advantage of this method lies in its ability to generate eccDNA fragments of different sizes from different genomic loci, including circular DNA molecules carrying intact genes, and it is applicable to various cell types. Moreover, this technique can also achieve targeted circularization of large genomic fragments or minichromosomes. However, the long-term stability of eccDNAs in mammalian systems remains unclear, and it is currently unknown whether these circular DNA molecules can replicate. Overall, CRISPR-C is a powerful and flexible experimental tool that can target and efficiently construct specific eccDNAs.

3) CRISPR/Cas9

Theoretically, it is feasible to reduce the copy number of eccDNA using the CRISPR/Cas9 system [126,127, 128]. This method aims to knock out eccDNA by specifically targeting its junction sites **(**Fig. 5**C).** Studies have shown that the eccDNA in maternal plasma exhibits distinct nucleosome − related characteristics in fragment size distribution. For example, there are significant peaks around 202 bp and 338 bp, showing a periodicity of approximately 10 bp. The 202 bp may correspond to the length of one nucleosome core plus two segments of linker DNA, while the 338 bp may correspond to the length of two nucleosome cores plus the linker region [92]. In addition, the analysis of common eccDNA junction sites in maternal plasma reveals that there are four types of enriched trinucleotide motifs around these sites, indicating a certain degree of sequence conservation at these circularization sites. Therefore, designing the CRISPR/Cas9 system based on these conserved sequence features to specifically target the junction regions of eccDNA for knockout or knockdown is a promising research direction.

4) CRISPR-hapC

CRISPR-hapC is a novel haplotype analysis method using CRISPR technology. It relies on CRISPR-induced knockout of chromosomal DNAs forming eccDNA in cells, enabling the joining of two distant genomic regions at their junctions **(**Fig. 5**D)**. The methodology encompasses several critical stages. Initially, in accordance with the CRISPR-C protocol, 6 eccDNA-targeting CRISPRs (designated as Chr1-ECC-Cr1 through Chr1-ECC-Cr6) were strategically designed in proximity to specific single nucleotide polymorphisms (SNPs). Specifically, Chr1-ECC-Cr1 is located upstream of SNP1, whereas Chr1-ECC-Cr2 through Chr1-ECC-Cr6 are sequentially positioned downstream of SNP2 through SNP6. This arrangement enables the production of eccDNA ranging from 660 base pairs to 211 megabases, effectively linking SNP1 with the other SNPs at their junctions. Following this, a pair of CRISPRs—Chr1-ECC-Cr1 and one of the remaining 5—are introduced into the cells via transfection to facilitate eccDNA formation through the CRISPR-C mechanism. CRISPR-C and CRISPR-HapC are unsuitable for in vitro eccDNA synthesis because they rely on live cell cultures and are typically used for larger eccDNAs [129].

CRISPR-hapC is a technology used for identifying and typing haplotypes, with a detection range spanning from several hundred bp to over 200 Mb. Advantages of this method include easy operation, high flexibility and low cost. However, it also has some limitations. The current version is based on the assumption that diploid cells contain only two haplotypes. Therefore, gene copy number variations (CNVs) or chromosomal polyploidy may affect this technology's application and results. Additionally, CRISPR-hapC is more effective at resolving the haplotypes of larger eccDNA fragments than shorter ones. It is also important to note that this technology relies on living cells (and their endogenous mechanisms) to function.

## Conclusion and prospect

Recent technological advancements in genomics and systems biology have catalyzed a transformative period in eccDNA research. Techniques such as clustered regularly interspaced short palindromic repeats (CRISPR-Cas9) genome editing and sophisticated cell imaging have facilitated the systematic exploration of eccDNAs [126,130,131]. As a novel category of genetic material, eccDNAs are characterized by their ubiquity, mobility, and functionality. EccDNA originates from different regions of the eukaryotic genome, and its functions depend on the types and combinations of genetic elements it carries, thereby exhibiting diversity and specificity in processes such as gene regulation, cancer progression, and treatment resistance. For example, eccDNA carrying enhancers can activate gene transcription distally or proximally. Its circular structure endows it with high physical mobility, enabling it to mediate inter − chromatin interactions and participate in the formation of new regulatory networks. This structure also helps enhancers break free from the constraints of the original TADs, circumvent insulator suppression, re − activate silent enhancers through spatial reconstruction, and subsequently significantly up − regulate the expression of oncogenes. Additionally, eccDNA can aggregate to form “eccDNA hubs”, allowing multiple circular DNAs to be in close spatial proximity, acting synergistically and significantly amplifying oncogene transcription [43]. Due to its circular and free characteristics, eccDNA has the function of a “mobile enhancer” and can widely activate multiple genes across chromatin boundaries [46]. Some eccDNAs can serve as templates for the transcription of circRNAs or carry miRNA coding sequences to transcribe regulatory miRNAs that silence target mRNAs [82,132]. They can also transcribe siRNAs or carry lncRNA sequences, and their transcription products can participate in the regulation of tumor − related gene networks through mechanisms such as ceRNA. In summary, eccDNA plays an important role in gene expression regulation, but most of its specific mechanisms remain unclear. Currently, this field is still in its infancy, and further in − depth research is needed to systematically reveal its functional mechanisms.

eccDNA exhibits higher stability than linear DNA due to its unique ring structure, which effectively resists degradation by nucleic acid exonucleases 11,13. This property allows it to remain in the body for a longer period of time, is easier to detect, and is widely present in a variety of body fluids (e.g., serum, plasma, and urine) [11,62,133,134]. Therefore, eccDNA has become a promising biomarker in liquid biopsy and has received widespread attention in disease diagnosis and applied research. Studies have shown that the average length of eccDNA in preoperative serum and plasma is higher than that in postoperative period in the same patient; it has also been reported that eccDNA in human plasma is usually longer than that of linear DNA, and that maternally-derived eccDNA may be longer than that of fetal origin [10,11]. Notably, eccDNA detected in cancer tissues is also often longer than in normal tissues, a property that justifies its use as a noninvasive diagnostic tool for cancer [19]. Although the total concentration of eccDNA in plasma is much lower than that of linear DNA, its longer fragments may carry more complete genomic information, showing unique applications. In addition, eccDNA can be detected in the urine of both healthy people and patients with advanced chronic kidney disease, and its content may further increase with the progression of the disease, suggesting that urinary eccDNA has the potential for the diagnosis and monitoring of urologic diseases. Currently, the research in this field is still at an early stage, and a mature research system has not yet been established, which still faces many challenges. For example, how to efficiently and stably enrich eccDNA from body fluids, as well as how to develop new technologies and assays suitable for systematic screening of eccDNA, are key issues that need to be addressed.

The varied sizes and types of eccDNA may influence its preferred mode of release. Under physiological conditions (e.g., apoptosis) and pathological circumstances (e.g., necrosis), eccDNA can exit the nucleus and enter extracellular fluids like serum, plasma, and urine [13,54,135]. Directly traversing the cell membrane is challenging for eccDNA; hence, its release and intercellular transport likely involve specific mechanisms. For example, it can mediate immune responses through the cGAS — STING DNA − sensing pathway or be packaged into extracellular vesicles such as exosomes for active transport, thereby participating in intercellular communication [92,136]. Taking eccDNA released by apoptotic cells as an example, it can be naturally taken up by bone marrow − derived dendritic cells (BMDCs) into the cytoplasm. cGAS can strongly recognize such eccDNA — its circular conformation lacks DNA ends, which may make it easier to avoid degradation and effectively mimic the “U − shaped structure”, thus serving as a potent agonist for cGAS. After cGAS is activated, it synthesizes cGAMP, which in turn activates the STING–TBK1–IRF3 signaling axis. Phosphorylated IRF3, together with other transcription factors, initiates the transcription of type I interferons (such as IFN — α and IFN — β), as well as various inflammatory and chemotactic factors (such as ISGs). Activated BMDCs then secrete large amounts of type I interferons (such as IFN − α/β) and various cytokines (such as IL — 6, TNF — α, etc.). It is known that certain host factors (such as HMGB1 and TFAM) can enhance cGAS recognition of linear DNA by inducing DNA bending to form a U — shaped structure [92,137,138]. The inherent circular conformation of eccDNA may naturally mimic or even be superior to such “U — shaped structures”, making it a highly effective agonist for cGAS. Therefore, the cGAS–STING pathway is the core mechanism for recognizing intracellular eccDNA, amplifying signals, and ultimately mediating cytokine secretion into the extracellular environment. Extracellular vesicles (EVs) can mediate the intercellular transfer of mtDNA. For example, EVs derived from cancer − associated fibroblasts (CAFs) can transport mtDNA into dormant breast cancer stem cells (CSCs), promoting their exit from dormancy and enhancing resistance to endocrine therapy. This indicates that mtDNA plays an important signaling role in intercellular communication. Although current research mainly focuses on mtDNA carried by EVs (which is a type of eccDNA), other nuclear — derived eccDNAs may also be transferred through a similar vesicle — mediated mechanism. Many details and the universality of relevant research still need further experimental verification. In summary, the cross — cell regulation of eccDNA is a challenging yet promising research direction, and future studies are expected to provide new breakthroughs in the treatment of diseases such as cancer.

This paper thoroughly examines the potential functions and mechanism of action of eccDNA and posits several hypotheses. It systematically reviews established technologies and introduces novel perspectives in research methodology. Additionally, it anticipates future research trajectories in this domain, proposing new scientific inquiries and avenues for more comprehensive exploration. Despite encountering various challenges, research on eccDNA holds significant promise for future advancement.

## Compliance with ethics requirements

There were no experimental animal or human studies.

## CRediT authorship contribution statement

Hui Li, Yaling Chen and Jian Wang conceptualized and supervised the study. Xudong Liu and Xinxin Li contributed to the study design, Yaling Chen drafted the manuscript and prepared the tables and figures. Zhuanjian Li and Hui Li reviewed and prepared the final manuscript. All authors have read and approved the article.

## Declaration of competing interest

The authors declare that they have no known competing financial interests or personal relationships that could have appeared to influence the work reported in this paper.
